# Managing health worker migration: a qualitative study of the Philippine response to nurse brain drain

**DOI:** 10.1186/1478-4491-10-47

**Published:** 2012-12-19

**Authors:** Roland M Dimaya, Mary K McEwen, Leslie A Curry, Elizabeth H Bradley

**Affiliations:** 1 Philadelphia College of Osteopathic Medicine, Philadelphia, PA, USA; 2 State of Alaska Division of Public Health, Section of Health Planning and Systems Development, Juneau, AK, USA; 3 Yale School of Public Health, Division of Health Policy and Administration, Yale University, New Haven, CT, USA

**Keywords:** Nurse migration, Brain drain, Brain circulation, Human resources for health, Filipino nurses, Philippines

## Abstract

**Background:**

The emigration of skilled nurses from the Philippines is an ongoing phenomenon that has impacted the quality and quantity of the nursing workforce, while strengthening the domestic economy through remittances. This study examines how the development of brain drain-responsive policies is driven by the effects of nurse migration and how such efforts aim to achieve mind-shifts among nurses, governing and regulatory bodies, and public and private institutions in the Philippines and worldwide.

**Methods:**

Interviews and focus group discussions were conducted to elicit exploratory perspectives on the policy response to nurse brain drain. Interviews with key informants from the nursing, labour and immigration sectors explored key themes behind the development of policies and programmes that respond to nurse migration. Focus group discussions were held with practising nurses to understand policy recipients’ perspectives on nurse migration and policy.

**Results:**

Using the qualitative data, a thematic framework was created to conceptualize participants’ perceptions of how nurse migration has driven the policy development process. The framework demonstrates that policymakers have recognised the complexity of the brain drain phenomenon and are crafting dynamic policies and programmes that work to shift domestic and global mindsets on nurse training, employment and recruitment.

**Conclusions:**

Development of responsive policy to Filipino nurse brain drain offers a glimpse into a domestic response to an increasingly prominent global issue. As a major source of professionals migrating abroad for employment, the Philippines has formalised efforts to manage nurse migration. Accordingly, the Philippine paradigm, summarised by the thematic framework presented in this paper, may act as an example for other countries that are experiencing similar shifts in healthcare worker employment due to migration.

## Background

The Philippines has emerged as a leading labour-exporting country; according to the Commission on Filipinos Overseas (CFO), of the 9.4 million Filipinos living abroad as of 2010, 4.3 million Filipinos [1] are documented as living outside of the Philippines under temporary, work-related residence programmes. Accordingly, overseas Filipino workers (OFWs) represent major contributors to the Philippine economy through their remitted incomes, with US$ 20.1 billion remitted in 2011 [2], constituting 11% of the Philippine gross domestic product for that year [3]. Given the high demand for nursing professionals to fill overseas shortages, the nursing sector has been a major source of OFWs. From 2004 to 2010, nurses comprised an average of 19% of all emigrating Filipino professional, medical and technical workers [4]. As a result of this “nurse brain drain,” the Philippine healthcare system has experienced negative effects, demonstrated by numerous hospital closures and high nurse turnover [5,6].

Responding to such effects on employment, while maintaining perspective on the economic benefits of OFWs, representatives from labour, trade and healthcare have engaged in multifaceted policy dialogue. Coalescing as the Human Resources for Health Network (HRHN), this group of policy actors operates as a recommendatory body to the Philippine government, charged with developing national policy agendas and action plans [5]. With efforts like the institutionalization of the HRHN and other formal mechanisms to manage migration for employment, the Philippine model of policy development may offer guidance to other countries, such as India and Nigeria [7,8], that are emerging as nurse source-countries and may encounter similar shifts in workforce due to brain drain.

The goal of this study was to characterise the process by which Filipino nurse migration drives development of policies that address issues in employment, domestic healthcare and international cooperation. To achieve this goal, this study utilizes qualitative data from interviews with members of the HRHN and focus group discussions (FGDs) with Filipino nurses to create a thematic framework that: (1) highlights the impact of brain drain on nurse education and employment; (2) identifies the key elements of the process by which responsive policy is developed; and (3) conceptualises the perceived outcomes of future policy implementation.

## Methods

### Data collection

In-depth interviews were conducted in person with 10 key informants in Manila, Philippines. These informants were selected based on their participation in the HRHN and their high level positions within public and private entities involved in nurse education, employment and migration (Table 1). Upon discussing the study and non-disclosure of their institutional positions within the study, all 10 informants agreed to participate.

**Table 1:** Key informant interview participant affiliations

**Institution**	**Division/Department**
Association of Deans of Philippine Colleges of Nursing (ADPCN)	Executive Board
Association of Nursing Services Administration of the Philippines (ANSAP)	Executive Board
Commission on Filipinos Overseas (CFO)	Policy, Planning, and Research Division
Commission on Higher Education (CHED)	Technical Panel on Nurse Education
Department of Health (DOH)	Health Human Resources Development Bureau (HHRDB)
Philippine Nurses Association (PNA)	Executive Board
Philippine Overseas Employment Administration (POEA)	Employment and Welfare Office
Professional Regulation Commission Board of Nursing (BON)	Board of Nursing Officers

FGDs were held in a range of hospital settings with nurses of varying employment positions to adequately capture a sample of diverse perspectives. After receiving a description of the current study, nurse managers at selected hospitals recruited participants for the FGDs. Upon receiving a description of the study, all 58 nurses agreed to participate. Six FGDs were conducted, each consisting of 8 to 11 participants. Five FGDs were conducted at tertiary care hospitals in Manila and one FGD was conducted at a tertiary care hospital in the Ilocos Norte province (Table 2).

**Table 2:** Characteristics of focus groups

	**Hospital location**		**Hospital type**		**Nurse position**			**Participant gender**	
	Urban	Province	Public	Private	Volunteer	Staff	Senior-staff	Female	Male
A	×		×		×			5	3
B	×		×		×			6	4
C		×	×				×	8	2
D	×			×		×		7	2
E	×			×		×		10	1
F	×		×				×	9	1

Between June and August 2010, the first author conducted all 10 key informant interviews and moderated all six FGDs. Interviews and FGDs were carried out in English and used an interview guide (Additional file 1: Appendix) that questioned participants on their perceptions of the effect of nurse brain drain on education and employment in the Philippines and responsive policies and programs. Given the semi-structured format of the conversations, discussions varied based on the informant or focus group, with probing of individuals’ responses to elicit rich qualitative data. Interviews and FGDs were audio-recorded, independently transcribed and reviewed for accuracy.

### Data analysis

Coding and analysis of data were performed by the authors, representing diverse backgrounds in global health, health policy and qualitative analysis. The code structure was developed inductively, using the qualitative data to identify recurring concepts. The team reviewed coded transcripts and negotiated any discrepancies in coding to refine the code structure until no new concepts emerged from the data. Using the final, revised code structure, all data were re-coded and a framework was developed to highlight key emerging themes (Table 3).

**Table 3:** Domains and key themes in perceptions of nurse migration as a driver of policy development

**Context of nurse migration**
Push and pull factors drive nurse migration
**Problems created by nurse migration**
Shifts in quality of student-nurses and nurse education
Shifts in quality and quantity of nurse labour supply
**Key elements in the policy development process**
A central advisory body guides policy development and advocacy
Development of professional norms unites the nursing sector
National policy and programme innovations reflect the dynamic nursing landscape
Transnational cooperation is integral for ethical recruitment of foreign nurses
**Perceived outcomes of policy-driven changes**
Domestic mind-shifts
Global mind-shifts

Audio transcripts were entered into Atlas.ti (Version 5.2.9; Scientific Software Development GmbH) to facilitate organisation and analysis of data. Throughout data coding and code structure development, the team utilised an audit trail to document analytic decisions. This study was approved by the Yale University Human Investigation Committee.

## Results

Four domains characterised participants’ perspectives on nurse brain drain and responsive policy: context of nurse migration; problems created by nurse migration; key elements of the policy development process; and perceived outcomes (Table 3). Within each domain, emergent themes further illustrated participants’ perceptions; these themes are presented here, with representative quotations from key informants and FGD participants.

### Context of nurse migration

#### Push and pull factors drive nurse migration

Participants described push factors as internal conditions within the Philippines that have pushed Filipino nurses to leave the country. Nurses ranked low salary, within both the public and private sector, as the main push factor. Poor working conditions, outdated healthcare technologies, and lack of employment opportunities were described as other key push factors. Recently hired nurses also described the social pressures rooted in familial expectations:

"“In the first place, I really did not like to take up nursing, but my parents encouraged me … so I can go abroad and send money to my family.”"

In comparison, pull factors were defined as characteristics of nurse-destination countries that attract nurses away from the Philippines, such as higher salaries, higher quality working conditions and technologies and job vacancies due to local shortages. In addition to these appealing labour conditions, migration is facilitated by destination countries’ visa provisions for family petitions, as explained by an administrator from the Commission on Filipinos Overseas:

"“The ‘holy grail’ for nurses is to get to the US, because you have the doctrine of family reunification [that] ensures that you can petition for your family members to join you once you work in the US … that’s one of the motivating factors for nurses to go to the US.”"

The US has historically established special visa programmes for foreign nurses to supplement domestic shortages. Many key informants referenced the US Nursing Relief for Disadvantaged Areas Act, introduced in 1999 and reauthorized in 2006, which allowed eligible, understaffed US hospitals to employ foreign registered nurses under the H1-C visa [9].

### Problems created by nurse migration

#### Shifts in in quality of student-nurses and nurse education

Driven by push and pull factors to work abroad, Filipinos have enrolled in nurse education programmes in droves, with matriculation rates closely following global demand patterns for nurses. Accordingly, student-nurses’ motivations have shifted away from care for the community and towards employment abroad. A senior staff nurse reflected:

"“We can’t find empathy and caring among these new nurses. There’s only one motivation for why they took nursing: to go abroad … not to deal with the patients, just to deal with dollars. It’s far different from the motivations that we had.”"

Given this high demand for training to work internationally, the Philippine nurse education system has also shifted to match destination countries’ preferences for clinical, specialty nurses. A senior staff nurse observed:

"“We’re losing community health nurses, they are more focused on the clinical settings like secondary and tertiary hospitals. Slowly, these nurses are losing interest in community health … because they don’t need it abroad.”"

Key informants referred to the “mushrooming” or the drastic increase, in number of nurse training programmes from approximately 40 in the 1980s to nearly 470 in 2010 [10]. Administrators from academic and professional nursing organisations characterized the quality of education at these recently established programs as low quality, citing constant underperformance on nurse licensure examinations. Key informants attributed the shortcomings to inexperienced faculty, inadequate facilities and decreased exposure to patient care, due to clinical training sites overcrowded with student-nurses.

#### Shifts in quality and quantity of nurse labour supply

Despite the decrease in domestic supply implied by the term “brain drain,” participants described the paradoxical Philippine phenomenon, in which brain drain has led to an oversupply of recent nurse graduates. As more student-nurses finish their coursework, they are not hired by foreign employers due to lack of professional experience, adding to the pool of unemployed nurses still seeking opportunities to gain specialty training in the Philippines. Nurses and administrators observed that as skilled nurses migrate abroad, the nurses that fill their vacant positions are those with limited postgraduate work experience. Key informants described that even despite this turnover, the pool of nurse graduates remains large. Using back-of-the-envelope calculations, an administrator from the Board of Nursing estimated between 60 000 to 70 000 tenured nursing positions in the country. Concerning unemployment, the administrator elaborated:

"“This is not very ideal for the Philippines because when the job market abroad opens, we may have a vacuum for those nurses occupying the 60–70 000 positions and what would be left for the Philippines are the 150 000 inexperienced and unemployed nurses.”"

FGD participants described two options for unemployed nurses: to seek employment in other sectors, such as retail work and outsourced callcentres, or to attempt to gain clinical experience as a “nurse-volunteer.” These volunteer programmes employ recent nurse graduates to carry out various nurse-related duties, without pay and with a buy-in fee for participation. A nurse-volunteer described her actual duties:

"“I was expecting that we would be exposed … in the ward, in the ICU. It was more on going out to other departments, giving out memos, photocopying papers and answering calls, simple errands.”"

Despite receiving little to no exposure to the clinical setting, nurse-volunteers described these programmes as advantage to their applications for work abroad, under the assumption that “any experience in the hospital is good experience”.

### Key elements in the policy development process

The following themes represent strategies that policymakers have employed in their response to the impact of nurse migration on education and employment. These elements demonstrate the process by which policies and programmes have been developed to respond to the unique problems created by the migration of Filipino nurses abroad.

#### A central advisory body guides policy development and advocacy

The Human Resources for Health Network (HRHN) was convened in 2006 as an advisory body, comprised of administrators from public and private sector organisations involved in healthcare, employment and migration (including, but not limited to, institutions listed in Table 1). Organised by the Department of Health (DOH), the HRHN is charged with implementing the DOH Human Resources for Health Master Plan, which provides guidance for policies and programmes aimed at further developing and regulating healthcare workers in the Philippines. To craft comprehensive policy directions, the HRHN developed an organizational structure utilizing technical working groups (TWGs). While the HRHN manages human resource development at multiple levels, the impetus for its creation was Filipino nurse brain drain, as described by an immigration administrator:

"“Out-migration in the nursing sector was one of the reasons the HRHN came up. And that’s why they started looking at interventions through entry, but it really sprang from the exit TWG. If you were here during the early stages, the theme was always how to address migration.”"

HRHN member institutions are organised into TWGs based on the scope of their jurisdiction. The TWG on entry encompasses policy and regulation of education and training for healthcare workers, the TWG on workforce covers issues of domestic labour and employment, and the TWG on exit focuses on ethical recruitment by foreign employers and transnational agreements with destination countries. An Oversight Committee, led by representatives from the DOH, reviews policy directions and advocacy programs developed by each TWG [11]. At the time of data collection, a Bill was undergoing Philippine Congressional approval, seeking to formalise the HRHN as the official advisory body on human resources in health development.

#### Development of professional norms unites the nursing sector

As the nursing arm of the Professional Regulation Commission, the Board of Nursing (BON) is responsible for monitoring and regulation of nurse licensure, in addition to establishing standards for the profession as a whole. In order to identify and coordinate key nursing bodies and professional nursing organisations, the BON spearheaded the creation of the Nurse Roadmap, as described by a BON administrator:

"“We agreed that we can unite the nursing profession through good governance among the coordinating bodies through the Nurse Roadmap. We have more than 20 nursing [organisations]. At each level, there are expectations and initiatives.”"

Administrators described the Roadmap as an initiative to establish accountability and coordination among all actors within the nursing sector, distributing responsibility and leadership among relevant bodies.

The Nurse Roadmap was also discussed as a set of professional norms, created with the goal of asserting the role and competencies of Filipino nurses in the Philippines and abroad. Educational administrators described that through revision of curriculum and education standards, efforts like the Nurse Roadmap address brain drain’s negative effects on student-nurses’ education and future quality of practice:

"“So we have a good curriculum, especially the latest one because we put in place all the guidelines not only for the classroom activities but also for the related learning experiences they would have. The competencies and the core values are spelled out.”"

#### National policy and programme innovations reflect the dynamic nursing landscape

Philippine policymakers have initiated innovative programmes in nursing care delivery with two goals: (1) to draw upon the pool of nurses that are unable to find work domestically or abroad and (2) to utilise these nurses’ skills in delivering healthcare to underserved and rural areas of the Philippines. Two recent programs were established through collaborations between the Department of Labor and Employment (DOLE), DOH, BON and local government units.

The Nurses Assigned to Rural Service programme, or NARS, was described as a stop-gap solution to unemployment through deployment of nurses to rural, underserved areas for a six-month commitment. In these areas, the nurses rotated between a hospital-based clerkship, with focus on gaining advanced clinical experience, and a community health clinic, where nurses developed and implemented health education initiatives. At the time of data collection, it was reported within the HRHN that a total of 11 085 nurses were deployed through the NARS program. Further quantitative assessment of the project was unavailable, but anecdotal evidence highlighted both public and professional response. While many key informants discussed the successful reception of community health nurses among the public and the increase in nurses’ exposure to public health, some nurse administrators commented on the six-month deployment period as inadequate to maintain sustainability.

Drawing on the positive response to NARS participants’ work in community and public health, Project EntrepreNurse was created to encourage nurses to practice independently as primary care providers through nurse-managed cooperatives. Based out of a community clinic, the cooperative provides onsite primary care services and deploys nurses to patients’ households to provide home healthcare. A BON administrator further described the project’s structure:

"“…in the cooperative, you will have nursing officers supervising clinic programs and care activities in many of the homes. So you convert the community into a hospital and we service the health needs of the population. The vision is to have one nursing clinic in every barangay, then that is primary level care.”"

With start-up funding and support provided by DOLE and through coordination with local *barangay*, or district, government units, the nurse cooperatives were designed to respond to local health needs and to implement relevant health education initiatives. With cooperatives as the focal point of Project EntrepreNurse, DOLE emphasised the training of nurses in management and business practices. At the time of data collection, the first nurse cooperative was being piloted in Davao City.

#### Transnational cooperation is integral for ethical recruitment of Filipino nurses

Key informants discussed the potential impact of the WHO Global Code of Practice on the International Recruitment of Health Personnel, adopted at the 2010 World Health Assembly, which had recently taken place in the weeks prior to data collection. Recognition of the Code through bilateral labour agreements was described as a way to ensure that destination countries’ recruitment does not produce or aggravate negative health or workforce outcomes in source countries.

Appealing to the WHO Code, policymakers have expanded the focus of bilateral labor agreements not only to emphasise the protection of Filipino nurses abroad, but also to include destination countries’ responsibility for human resource development in the Philippines. An administrator from the Philippines Overseas Employment Agency explained the idea of “brain circulation”:

"“We need to work together to manage the situation such that even if [nurses] leave, we will still be able to maintain good healthcare delivery services. Through ‘giving back’ mechanisms, we hope to receive forms of assistance to our healthcare sector by way of investment and training assistances, their experts and technologies can help upgrade our systems because many of our facilities lack modern equipment and techniques.”"

In addition to transnational exchange of support, nursing administrators and educators have sought to engage the expertise of Filipinos residing abroad through “reintegration” programmes. These initiatives would facilitate the return and temporary residence of Philippine-born or Philippine-descent nurses at educational institutions, where they would provide training support and workforce development. Nurse reintegration programmes were discussed as ways to not only encourage foreign-residing Filipino nurses to “give back” to their home country, but also as way to augment nurse education in the Philippines.

### Perceived outcomes of policy-driven changes

#### Domestic mind-shifts

Key informants and FGD participants discussed the Nurse Roadmap, NARS, and Project EntrepreNurse as efforts working towards two domestic mind-shifts: (1) definition of roles and responsibilities to strengthen the nursing sector and (2) assertion of nurses’ ability to practice independently as primary care providers.

The Nurse Roadmap was described to primarily function as overarching guidelines for nursing sector bodies to divide and delineate programmatic initiatives in training and care delivery. In addition to its goal of coordination and “good governance”, the inclusion of nurse competencies within the Roadmap also carries implications on the professional identity of nurses, which was described as abstract, as told by a staff nurse:

"“Unlike abroad, where there are definitions of what a nurse can do, I think the function of a nurse in the Philippines is much more limited … I think we need to establish a clear-cut definition of our independent function.”"

Through establishing and enforcing achievement of core competencies, as outlined by the Nurse Roadmap, nursing professionals and academic administrators pointed out the emphasis on internal improvement.

Whereas earlier policies responding to nurse migration focused on retention, members of the HRHN described a new approach: accepting nurse migration as an unavoidable pattern and developing policies to strengthen the workforce, internally, so that the Filipino nurse, regardless of employment abroad or in-country, could provide high-calibre patient care. Key informants defined the ideal “globally competitive nurse”:

"“When we say globally competitive, it does not mean we are automatically gearing them for an overseas labour market, but even in our own country, for us to be able to attract investments and make our employees employable, we have to strengthen our educational and training system.”"

The second mind-shift delves further into the nursing workforce, aiming to change Filipino nurses’ own perceptions of their capacities to apply their skills outside of the hospital setting. NARS and Project EntrepreNurse were developed to refocus nursing practice in the Philippines, from globally demanded hospital-based care to local independent practice, with nurses as primary care providers in the community. An administrator involved in Project EntrepreNurse described the larger goal behind the initiative:

"“It’s just a mindset, right? Unless the nurse is convinced that, on her own, she can hang her shingles, have her own business card, build her practice, then we’re really just employment driven. The nurses themselves need a large shift in mindset.”"

Administrators described that nurse-led initiatives, such as nurse cooperatives and nursing homes, would empower the nurse in their skills, while demonstrating the breadth of their capacity to practice healthcare and generate their own income, outside of the hospital. Nursing professionals also described further development of avenues for profitable independent-nurse practice as a means to increase nurse retention, in the sector and in Philippines.

### Global mind-shift

Bilateral labour agreements and utilisation of the WHO Code were discussed in context of achieving ethical recruitment of nurses through brain circulation. Nursing professionals described that brain circulation provides benefits for both the Philippines and nurse-destination countries. The Philippines profits from new technologies and advanced trainings as part of the “giving back” mechanisms. In providing these mechanisms, destination countries are benefitting by ensuring that Filipino nurses, whom they hire in the future, are trained adequately.

In addition to reciprocal trade and international cooperation, brain circulation also enables a mind-shift among destination countries, in which their roles and responsibilities as an active player in labour migration is clearly outlined. An academic administrator described:

"“It’s not a 2-D problem … [destination countries] may think that it’s just the usual one-way migration, but they have to understand that their policies actually drive the effects and results in the Philippines … they need to see their role in it.”"

Many key informants described Filipino nurse migration as an issue that has evolved from a domestic crisis of migrating professionals into an international exercise in supply and demand.

## Discussion

The findings of this study indicate that Filipino policymakers have considered the ramifications of nurse brain drain and are crafting policies that respond to key issues in education, employment and international relations.

Internal strengthening of the nursing sector through stricter regulation and establishment of standardized competencies provides a solution to key problems that have arisen from nurse brain drain. With the “mushrooming” of degree-granting nursing programmes that have consistently underperformed on licensure examinations, the universalisation of competencies allows for a standard against which all graduating nurses must be qualified. Presuming that such competencies translate into better nursing practice, coupled with new initiatives to employ nurses in underserved areas, the quality and scope of patient care may also improve.

Through active use of the WHO Global Code of Practice on the International Recruitment of Health Personnel in their forging of bilateral labor agreements, Filipino policymakers and their push for ethical recruitment are supported by an internationally recognised doctrine. Engagement with destination countries through “giving-back” mechanisms allows the Philippines to strengthen the nursing workforce externally, while also allowing destination countries to protect their future investments of Filipino nurses. By engaging in discourse on mechanisms of international exchange, the brain drain phenomenon is framed as an issue of transnational trade, with source countries and destination countries compelled to treat Filipino nurses and their skills as protected commodities.

### Limitations of this study

The qualitative nature of this study allowed for development of a thematic framework grounded in the perspectives of stakeholders involved in nurse migration. A common limitation of qualitative studies is the generalisability of the findings to the larger population, given purposive sampling of key informants and a small number of FGD participants, relative to the current stock of Filipino nurses. For broader perspective on the Philippine nurse population, future study should utilise samples of administrators and nurses from primary and secondary care centres in rural provinces. Lastly, in using qualitative methodology, this study elicited nuances within participants’ perspectives that cannot be statistically tested. This not only necessitates future quantitative approaches, but also long-term follow-up and evaluation of policy efforts discussed in this paper.

## Conclusion

As the Philippines continues to respond to problems of ongoing nurse migration, the thematic framework presented in this paper may act as a snapshot of the policy development process, for possible use in future evaluation and setting of policy directions. As other countries begin to experience similar patterns of migration for employment, contextual drivers and resulting problems may resemble those of the Filipino nurse brain drain phenomenon. Given such similarities, the Philippine paradigm characterised in this paper, may offer guidance in predicting the effects of long-term healthcare worker brain drain. Furthermore, understanding the Philippine policy development process may help to shape preventive measures to avoid declines in quality of nurse education or practice in other countries that are emerging as sources of nurses migrating for employment.

## Competing interests

The authors declare that they have no competing interests.

## Authors’ contributions

RD conducted in-country data collection, reviewed literature, conducted study design and data analysis, and drafted and revised the manuscript. MM conducted data analysis and drafting and revision of the manuscript. LC and EB participated in study design, data analysis, and edited the manuscript. All authors read and approved the final manuscript.

## Supplementary Material

Additional file 1 Appendix. In-depth interview and focus group discussion interview guide. Click here for file
