# Supplementary material for: Managing health worker migration: a qualitative study of the Philippine response to nurse brain drain
Source: Hum Resour Health. 2012 Dec 19;10:47. doi: 10.1186/1478-4491-10-47 (PMC3541120; doi:10.1186/1478-4491-10-47)
Supplement: Additional file 1 — Appendix. In-depth interview and focus group discussion interview guide. [file 1478-4491-10-47-S1.doc]

Appendix

In-depth interview and focus group discussion interview guide

In-Depth Interview Guide

1 Tell me about the current state of the nurse workforce and migration.

2 How does nurse migration affect other sectors? (Probe to elaborate on specific sectors, especially economy, education, and health.)

3 What policy actions do you think can be implemented to improve nurse education and the Philippine nurse ‘brand’?

4 Tell me what you know the NARS program and its goals. What impact will it have on rural health and the nurse workforce?

5 What strategies do you think would increase access to healthcare in rural areas? Which sectors must be involved to achieve this goal?

6 I’m trying to understand the effectiveness of policy in managing the effects of nurse migration; what other aspects of policy and implementation should I consider to understand the process more fully?

Focus Group Discussion Guide

1 Tell me what you know about changes to the nursing school curriculum.

2 What are your thoughts on the quality of nurse education in the Philippines? How do you think this reform will impact the quality of nurse education?

3 How can nurse education, curriculum, and administration be adapted to improve its quality? What types of training were missing in your own education that would have helped you in your current practice as a nurse?

4 Tell me about your experience in your current position. How would you characterize staffing, your own workload, and overall working conditions at your current job?

5 Tell me what you know about the NARS program. What impact will it have on rural healthcare?

6. What strategies can be employed to improve access to care in rural areas? How can nurses aid in these efforts?
